# A novel three-dimensional strapping reduction for the treatment of patellar fractures

**DOI:** 10.1186/s13018-019-1294-7

**Published:** 2019-08-06

**Authors:** Wei Jiang, Yusheng Li, Ronak Naveenchandra Kotian, Bowen Lin, Xiaoming Zhang

**Affiliations:** 1 Department of Bone and Joint, Shenzhen People’s Hospital, The First Affiliated Hospital of Nanfan University of Science and Technology, The Second Clinical Medical College of Jinan University, Shenzhen, 518020 People’s Republic of China; 2 Department of Orthopedics, Xiangya Hospital, Central South University, Changsha, 410008 People’s Republic of China; 3 National Clinical Research Center for Geriatric Disorders, Xiangya Hospital, Central South University, Changsha, 410008 People’s Republic of China; 4 Department of Orthopaedic Surgery, Victoria Hospital, Bangalore Medical College and Research Institute, Bangalore, India

**Keywords:** Patellar fracture, Three dimension, Reduction

## Abstract

**Objective:**

This study aimed to investigate the effectiveness of a three-dimensional strapping reduction in the treatment of patellar fractures.

**Methods:**

Between January 2015 and June 2017, a total of 56 patients were randomly allocated to the three-dimensional strapping reduction group (trial group) and towel clamp reduction group (control group). There were no significant differences in age, gender, injury side, the interval time from injury to surgery, fracture pattern, and cause of injury (*P* > 0.05). The operation time, fluoroscopy time, bone union time, postoperative Hospital for Special Surgery (HSS) scores, and complications were recorded and analyzed.

**Results:**

All incisions achieved primary union. All patients in both groups completed a follow-up with an average of 12.5 months (range 11–15 months). Both operation time and fluoroscopy time in the trial group were significantly shorter than those in the control group (*P* < 0.001). All patellar fractures achieved bone union, and there was no significant difference in bone union time between the two groups (*P* > 0.05). Bone nonunion, infection, and fixation failure were not found in both groups. HSS scores of the trial group (90.9 ± 4.2) were higher than those of the control group (86.6 ± 5.2) (*P* < 0.01).

**Conclusion:**

Compared with towel clamp reduction, the three-dimensional strapping reduction in the treatment of patellar fractures has advantages of shorter operation time and fluoroscopy time, better knee function after surgery, and satisfactory fracture healing.

## Introduction

Patellar fractures are common fracture type clinically, accounting for approximately 1% of the all human fractures, which are often caused by high-energy trauma. The knee function is usually severely compromised due to the destruction of the knee extension device; then, operative intervention is required to achieve a stable anatomic reduction of the patella [1–4]. Anatomic restoration of intact patellar articulation surface is essential to patellar fractures to avoid the incidence of patellofemoral joint traumatic arthritis [5–7]. At present, the conventional surgical method of patellar fractures is to reduce patellar fractures with the assistance of towel clamps. In this conventional method, anatomical reduction of the patellofemoral joint surface cannot be confirmed, because of limited operative field of the posterior surface of patellofemoral joint, especially for comminuted patellar fractures. Therefore, there is a high risk of malunion of patellar fractures and traumatic arthritis for patellar fractures [8, 9]. Based on aforementioned reasons, a novel three-dimensional strapping reduction was designed in the treatment of patellar fractures. We aimed to investigate the effectiveness of a three-dimensional strapping reduction in the treatment of patellar fractures, compared with the conventional method.

## Materials and methods

### General information

From January 2015 to June 2017, a total of 56 eligible patients with patellar fractures were included in this study. Exclusion criteria include pathological fractures, osteoarthritis, rheumatoid arthritis, or complicated with other fractures. According to the admission date, all patients were divided into the control group (towel clamp reduction group) and the trial group (three-dimensional strapping reduction group). This study was approved by the institutional ethical review board. All written informed consents were obtained before surgery.

In the trial group, there were 15 males and 13 females with an average age of 39.6 years old (range: from 20 to 65 years old). Based on the injured side, there were 12 patients with injury on the left knee and 16 on the right knee. The mechanism of patellar fractures was divided into fall (19 cases) and traffic accident (7 cases), and all fractures were closed fractures. The interval from injury to surgery ranged from 2 to 9 days (mean 4.7 days).

In the control group, there were 14 males and 14 females with an average age of 37.8 years old (range: from 21 to 66 years old). Based on the injured side, there were 11 patients with injury on the left knee and 17 on the right knee. The mechanism of patellar fractures was divided into fall (18 cases) and traffic accident (10 cases), and all fractures were closed fractures. The interval from injury to surgery ranged from 1 to 9 days (mean 4.2 days).

The demographic characteristics between these two groups were comparable including gender, age, injury side, injury mechanism and type, fracture type, and the interval from injury to surgery (*P* > 0.05).

### Surgical procedures

All operations were performed by the same surgeon in both groups.

#### Trial group

**Fig. 1:**
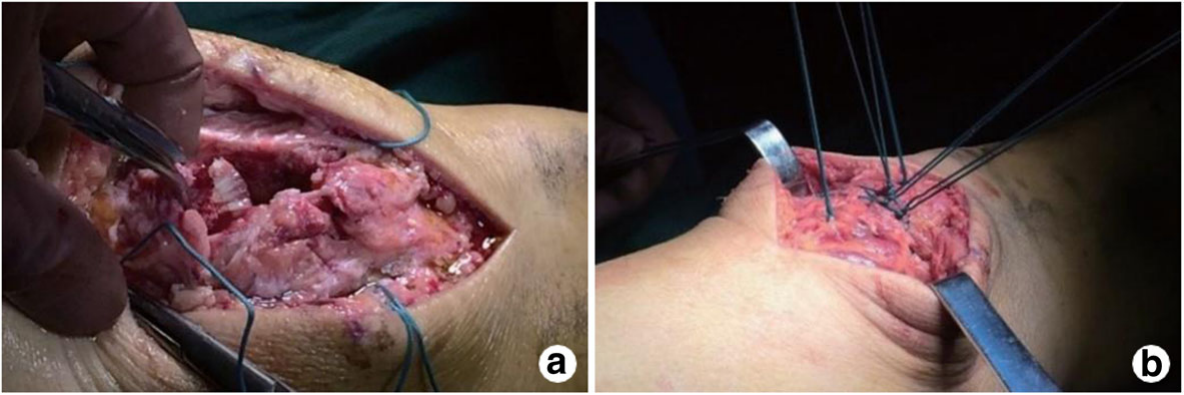
Operative procedures of three-dimensional strapping reduction. **a** The sutures threading from the fracture end to soft tissue around the patella through the patellofemoral joint. **b** All sutures tied up on the anterior surface of the patella after strapping reduction of the patella under direct vision

All patients were placed in a supine position under spinal anesthesia. The fracture site was exposed after removal of hematoma and injured soft tissue through a vertical incision in the middle of the patella. Then, the number of fracture line of the patella was observed and recorded. Generally speaking, the number of sutures used is equal to the number of major fractured bone. For example, two-part fractures are usually tied with two sutures to reduce and three-part fractures are treated with three sutures. The sutures (1-0, ETHIBOND, JIANGSU CAICHANG PHAMACEUTICAL CO. LTD) were threaded from the fracture end to soft tissue around the patella through the patellofemoral joint. Subsequently, all sutures were tightened and tied up on the anterior surface of the patella after the reduction of the patella under direct vision, and then, a satisfactory patellofemoral joint surface needed to be confirmed. A C-arm X-ray fluoroscopy machine was used to have further confirmation of anatomical reduction of the patellofemoral joint surface. Next, the strapping sutures were removed after application of tension band with knee flexion of 70°. Regarding comminuted patellar fractures, Herbert nails were used to provide a further fixation of a large fragment of the patella (Fig. 1).

#### Control group

There were no differences in anesthesia method, surgical position, approaches, and exposure method of fracture site between the control group and the trial group. The patellar fracture was reduced and fixed by two or three towel clamps after clearance of soft tissue around the fracture sites. Then, a C-arm X-ray fluoroscopy machine was used to confirm whether an anatomic reduction of the patellofemoral joint surface was achieved. The towel clamps were loosened, and the reduction was re-adjusted until a satisfactory reduction was obtained if the fracture reduction of the patella was unsatisfactory. Herbert nails were also used to fixed a large fragment of the patella for comminuted fracture before fracture reduction with the assistance of towel clamps. Finally, the tension band was used for final fixation of the patella.

### Postoperative rehabilitation and effectiveness evaluation

Quadriceps function exercise was initiated immediately after surgery. Continuous passive movement of flexion of the knee was performed to achieve a knee flexion of 90° and full weight-bearing walking within 2 weeks postoperatively. Normal passive flexion and extension of the knee were obtained 4 weeks postoperatively, and active flexion and extension of the knee were achieved 8 weeks postoperatively.

Operative time, intraoperative fluoroscopy time, and complication rate were recorded in this study. Bone union time was also recorded during follow-ups. The knee function was evaluated by Hospital for Special Surgery (HSS) scores.

### Statistical analysis

Statistical analysis was performed using the SPSS statistical version 19.0 in this study. Data was shown as mean ± SD. The student *t* test was used for continuous variables. *P* < 0.05 was regarded as a significant difference.

## Result

**Fig. 2:**
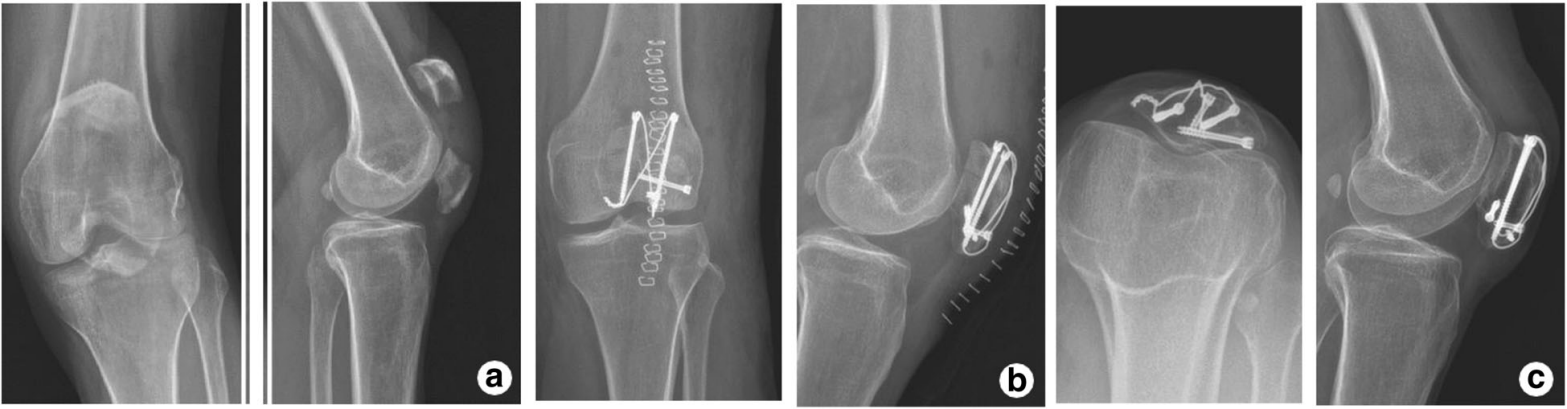
X-ray films of a 57-year-old female patient with comminuted fracture of the left patella in the trial group. **a** Before operation. **b** Immediately after operation. **c** At 1 year after operation

**Fig. 3:**
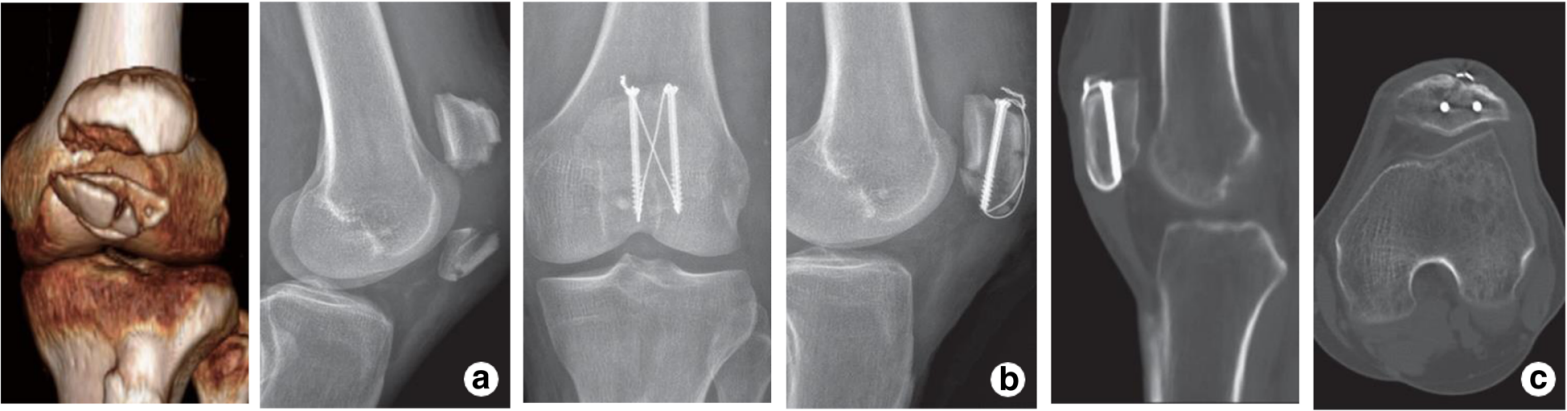
A 53-year-old male patient with comminuted fracture of the left patella in the control group. **a** CT and lateral X-ray film before operation. **b** Anteroposterior and lateral X-ray films immediately after operation. **c** Lateral X-ray films and CT at 1 year after operation

All incision achieved primary union. All patients completed the follow-ups with an average of 12.5 months (range 11–15 months). The operative time and intraoperative fluoroscopy time were 41.5 ± 6.9 min and 14.9 ± 3.1 min respectively in the trial group, compared to 60.1 ± 8.4 min and 22.6 ± 5.4 min in the control group. Both operative time (*P* < 0.001) and intraoperative fluoroscopy time (*P* < 0.001) significantly decreased in the trial group. All patients achieved bone union in both groups. There was no significant difference in bone union time between the control group and the trial group. Complications were not observed in both groups, such as bone nonunion, infection, and implant failure. At 6 months postoperatively, the HHS score was statistically higher in the trial group (90.9 ± 4.2) than in the control group (86.6 ± 5.2) (*P* < 0.005) (Figs. 2 and 3).

## Discussion

Patellar fractures account for around 1% of all human body fractures, commonly in adults of 20–50 years old, with a male/female sex ratio of 1:2 [3]. The majority of patellar fractures are caused by direct violence, and the most common indirect injury mechanism was eccentric contraction of the quadriceps muscle [2, 4]. As the largest sesamoid bone of the body, the patella plays an essential role in the resistance of knee flexion and extension, the patella also converts tensile forces into compression forces. Moreover, an intact patella and patellofemoral joint surface are essential for normal function of the knee. Therefore, the primary goal of treatment of patellar fractures is to restore an intact extension device and patellofemoral joint surface, henceforth to avoid the occurrence of traumatic knee arthritis [5–7, 10].

Clinically, stable nondisplaced patellar fractures (displacement < 2 mm) can be treated by the conservative method. Unstable patellar fractures however require surgical treatment. Tension band technique is the gold standard for surgical treatment of patellar fractures [11–14], with which patellar fracture reduction can be achieved by paralleled Kirschner wire and single strand “8” shaped wire fixation. There are complications such as soft tissue irritations and wire loosening. Thus, this technique was modified in recent years. Non-absorbable polyester braided sutures are also applied to instead of wiring cable [15–18]. In addition, the hybrid technique of cannulated screws and tension bands is proposed, two cannulated screws instead of Kirschner wires were inserted through by modified tension band wiring. This modified technique was regarded to have advantages to prevent the displacement of fracture fragments [19–21]. Subsequently, the technique of percutaneous cannulated screw and tension band was performed. The minimal invasiveness can reduce damage to the blood supply of patellar fracture fragments, which also make arthroscopic treatment of patellar fractures as an alternative [22]. The newly designed low profile mesh plates is also a novel technique for the treatment of patellar fractures [23–27].

Regardless of fixation methods of patellar fractures, it is essential to reconstruct anatomic reduction of the patella and restore the intact patellofemoral joint surface so that the incidence of traumatic knee arthritis can be reduced. Currently, patellar fracture reduction by towel clamps is the most common treatment of patellar fractures, but there existed several drawbacks. Firstly, whether the posterior joint surface achieved satisfactory reduction cannot be confirmed, although the anterior surface of the patella had an anatomic reduction with the assistance of towel clamps under direct vision. Secondly, the unique anatomy of the patella leads to difficult reduction of patellofemoral joint surface. When the anterior surface of the patella achieves a satisfactory reduction, there is usually a gap on the posterior surface of the patellofemoral joint. Thirdly, tension band should be inserted after fracture reduction by towel clamps, which may affect surgical procedure and intraoperative fluoroscopy, and even lead to reduction loss. Finally, about comminuted patellar fractures, there are few solid reduction sites of the patella for towel clamps, and the reduction of patellar fracture cannot be performed effectively. Hence, complications like bone malunion of the patella and incidence of traumatic knee arthritis are commonly reported [8, 9, 28].

Based on the facts mentioned above, we put forward a novel three-dimensional stapping reduction technique. The sutures were threaded from the fracture end to soft tissue around the patella through the patellofemoral joint; then, sutures were tightened and tied up one by one. The fracture fragment of the patella could be bundled by sutures from the posterior outwards three-dimensionally. Compared with the conventional fracture reduction technique with towel clamps, there were several advantages with this novel technique. Firstly, this technique could be applied to all fracture type of the patella, especially for comminuted patellar fractures. Secondly, this technique has a shorter operative time and easier approach. Thirdly, three-dimensional suture bundle of the patella from the posterior outwards contributed to better compliance. Concentric stress provided by sutures results in a more stable reduction of the patella. Fourthly, the knee can be flexed and extended without limitation after strapping reduction of the patella, which can simplify subsequent operation. Finally, intraoperative fluoroscopy was not affected. In the present study, compared with the conventional technique, three-dimensional stapping reduction of the patella fractures can shorten the operative time, decrease intraoperative fluoroscopy time, and contribute to the satisfactory functional outcome.

There are several limitations in this study. The theory of this technique and standard procedure need to be further classified. Furthermore, biomechanics analysis of this three-dimensional stripping technique is needed. A long-term large-sized prospective randomized controlled trial can better evaluate the efficacy of this technique.

## Conclusion

Compared with towel clamp reduction, the novel three-dimensional strapping reduction in the treatment of patellar fractures has several advantages: (1) shorter operation time, (2) less fluoroscopy time during operation, and (3) better knee function after surgery. Therefore, we recommend this technique for the treatment of patellar fractures.

## Data Availability

All the data and materials can be found in the manuscript.

## Abbreviation

HSS: Hospital for Special Surgery
